# Results of a New Correction Technique in Surgical Treatment of Idiopathic Scoliosis: Mid-term Clinical and Radiological Outcomes

**DOI:** 10.7759/cureus.3454

**Published:** 2018-10-15

**Authors:** Baver Acar, Ali K Us

**Affiliations:** 1 Orthopaedics and Traumatology, University of Health Sciences, Antalya Education and Research Hospital, Antalya, TUR; 2 Orthopaedics, Ankara University School of Medicine, Ankara, TUR

**Keywords:** correction technique, ilizarov wire, scoliosis, idiopathic, fusion

## Abstract

Introduction

The term "scoliosis" is defined as a spinal curvature of >10˚ in the coronal plane. A currently widely used method in scoliosis treatment is posterior instrumentation and fusion following deformity correction made with pedicle screws and rods. Various methods have been described for deformity correction during surgery, and each method has its own advantages and disadvantages. In 2009, Us et al. described a new correction method using an Ilizarov wire. The aim of this study was to present the mid-term results of the patients operated with this technique.

Methods

This study included 18 patients diagnosed with adolescent idiopathic scoliosis between 2006 and 2010, who underwent posterior instrumentation and fusion surgery with the intra-operative temporary traction method from the posterior.

Results

Based on the standing anteroposterior radiographs taken preoperatively, the Cobb angle of the major curvatures was calculated as mean 50.83˚ (range: 30˚-72˚). Postoperatively, the mean 68.8% correction at 15.77˚ was determined. At the final follow-up examination, a correction loss of mean 3.3% (range: 0% to 6.8%) was observed.

Conclusion

This technique can be considered a simple and safe alternative method for correction in scoliosis surgery.

## Introduction

Although the term "scoliosis" is defined as a curvature of the spine of >10˚ in the coronal plane, the deformity in scoliosis is not restricted to the coronal plane but may occur as a three-dimensional deformity, including the sagittal and axial planes. This deformity includes a lateral shift in the frontal plane, rotation in the axial plane and intervertebral extension, causing lordosis in the sagittal plane [1-2].

Parameters such as the degree of curvature, the localisation of curvature, patient age and gender affect the selection of conservative or surgical treatment methods in the treatment of scoliosis. The aim of scoliosis treatment is the clinical and radiological correction of the deformity, stabilisation, prevention of progression and providing and maintaining the balance of the vertebral column [1-3].

Currently, the widely used method for scoliosis treatment is posterior instrumentation and fusion following correction of deformity using pedicle screws and rods. Various methods have been described for deformity correction during surgery, and each method has its own advantages and disadvantages [4-8]. In 2009, Us et al. described a new correction method using an Ilizarov wire. The aim of this study was to present the mid-term results of patients operated with this technique [9].

## Materials and methods

This study included 18 patients diagnosed with adolescent idiopathic scoliosis between 2006 and 2010, who underwent posterior instrumentation and fusion surgery with the intra-operative temporary traction method from the posterior. The clinical and radiographic results of these patients were obtained preoperatively, immediately after surgery and at the final follow-up examination, and a retrospective evaluation was made. The Scoliosis Research Society form (SRS-30) was completed by the patients at the final follow-up examination, and the results were evaluated.

Preoperatively, the curvatures were classified using the King classification system and the Lenke classification system. Postoperatively, the Cobb angle was measured at the same levels on the anteroposterior and lateral radiographs taken in the early period. The correction rate in the coronal plane was calculated using the following formula:

Correction Rate (%) = [(Preoperative Cobb angle – Postoperative Cobb angle) / Preoperative Cobb angle)] x 100

The correction loss was evaluated on the anteroposterior and lateral radiographs taken at the final follow-up examination, and the percentage was calculated using the following formula:

Correction Loss (%) = [(Cobb angle at the final follow-up examination – Postoperative Cobb angle) / Preoperative Cobb angle] x 100

Surgical technique

**Figure 1:**
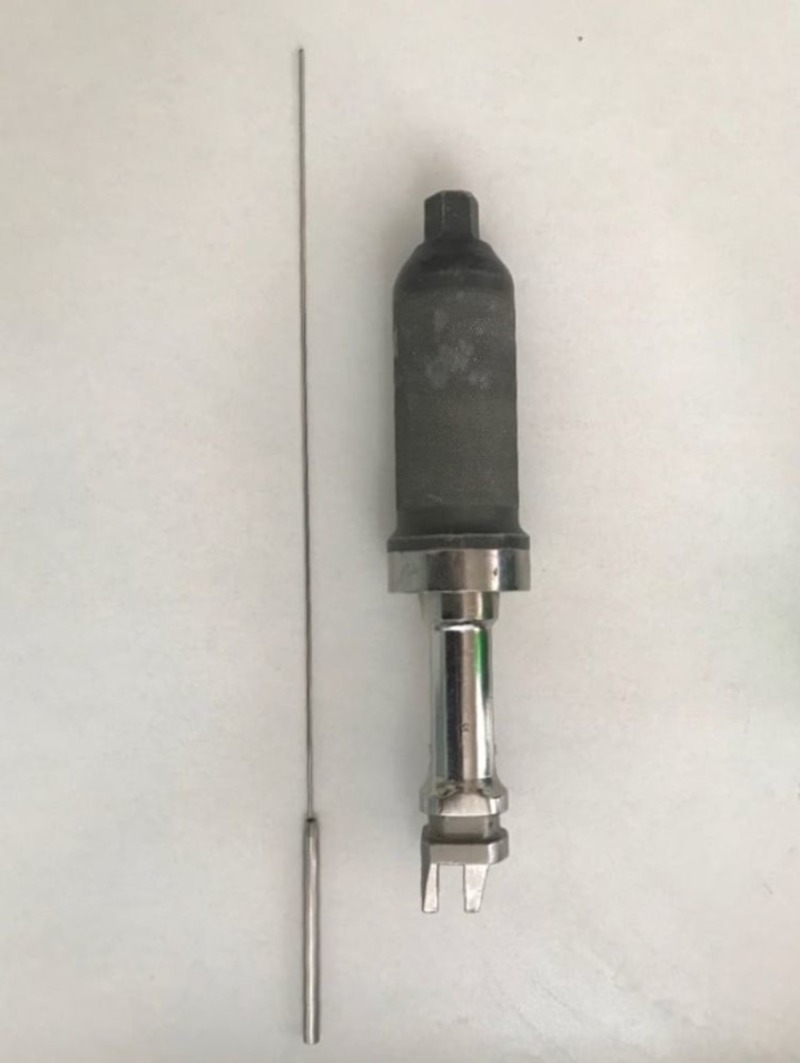
Technical equipment A specially manufactured K wire that has a wider end and Ilizarov K wire tensioner.

The Ilizarov traction device and specially designed Ilizarov wire of the thickness of the distal end rod were used in this method (Figure 1).

**Figure 2:**
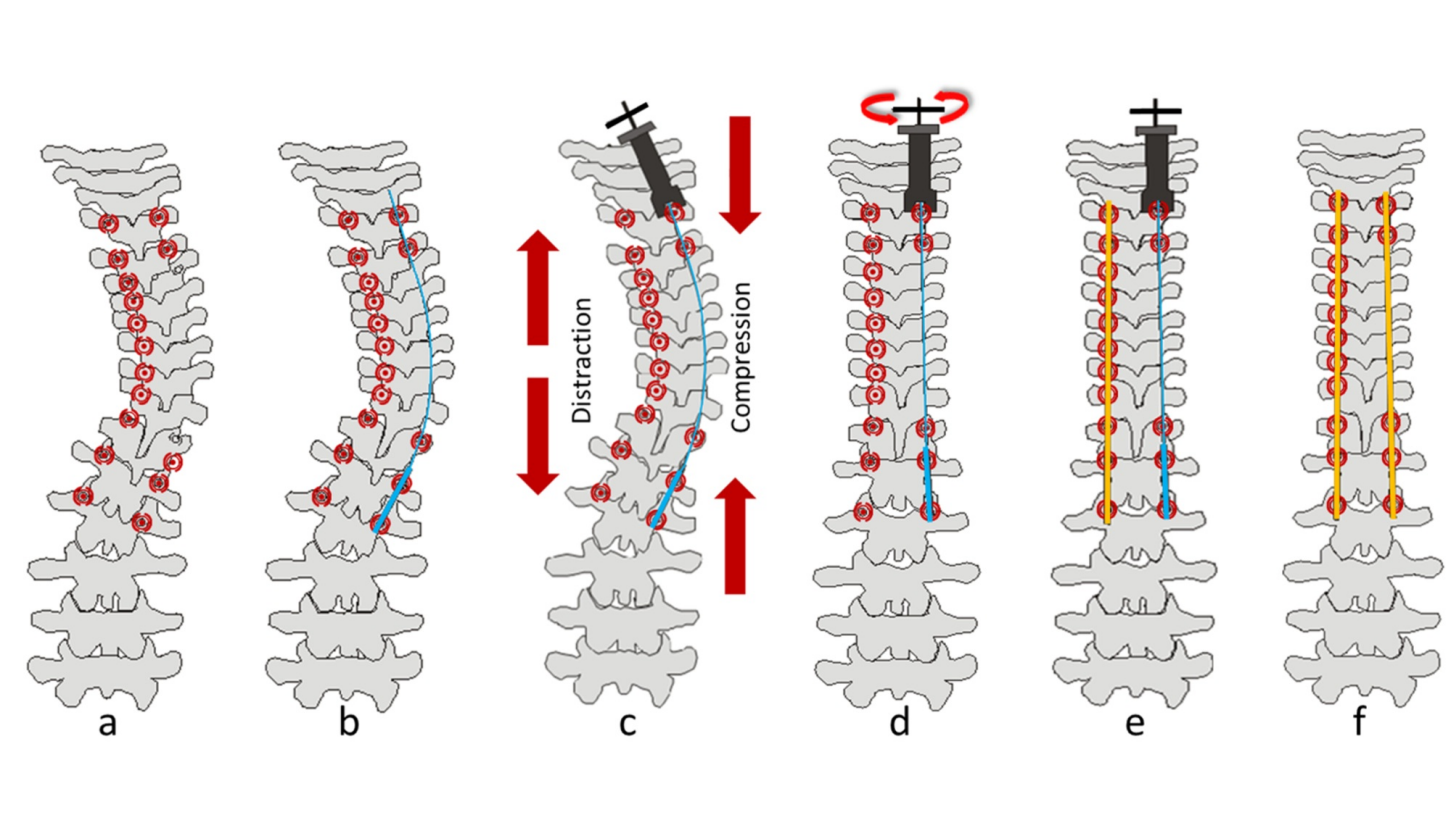
Technical steps Illustration showing the transient tension technique. (a) First, all pedicular screws on the convex side and pedicular screws on the concave most caudal and cranial 2-3 levels are placed. (b) A specially manufactured K wire that has a wider end is placed on the convex side through the screws and the rod is locked. (c) Afterward, the Ilizarov K wire tensioner is placed to the K wire and compression is applied. (d) Tensioning of the K wire is continued until the desired correction is achieved. (e) After the correction of deformity, the convex side rod is placed and locked. (f) Finally, the concave side rod is placed and the system is locked.

Transpedicular screws were placed on the convex and concave sides of the vertebrae with the scoliosis deformity using the standard method. The end of this wire of rod thickness was placed and locked within the distal or proximal screws on the convex side of the curvature. The wire section was passed through the intervening screws, and the imbus screws were placed over them (Figure 2).

**Figure 3:**
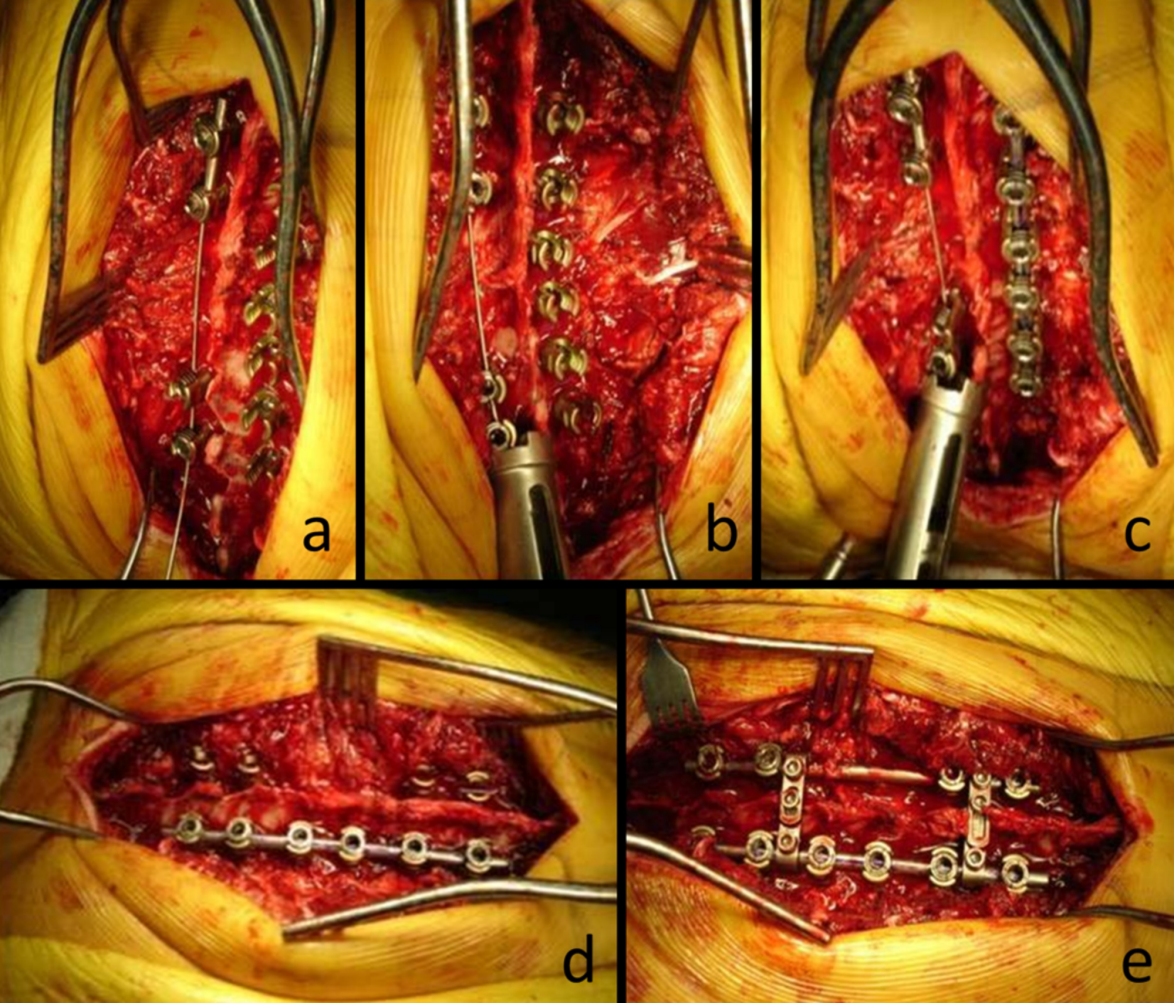
Clinical application of the technique Clinical application of the technique. (a) The K wire with a wider end is placed and locked. (b) The Ilizarov tensioning apparatus is placed, and deformity is corrected with compression. (c) Under the tension, the convex side rod is placed and locked. (d) The K wire is released and taken off. (e) Finally, the second rod is placed on the concave side and the system is locked with transverse connectors.

After taking the free end of the wire outside, the Ilizarov traction device was placed over the wire and drawn close to the screw. By turning the Ilizarov traction device slowly and in a controlled manner, the traction procedure was applied between the proximal and distal regions on the convex side until the desired correction was obtained in the deformity. When the desired correction was obtained, the concave side rod was bent to an appropriate curve and placed within the screws (Figure 2). If rotation was not corrected by a sufficient amount, the rod on the concave side was placed and locked by applying the de-rotation maneuver. The Ilizarov traction device and special wire were then removed. The rod on the concave side was placed and locked (Figure 3).

In this technique, the classic Ilizarov traction device was used, and with each quarter turn, up to approximately 1-mm traction force was applied. When fully compressed, seven full turns can be made and there can be up to 30-mm traction.

## Results

In total, 18 patients comprising 14 females and four males with a mean age of 16.05 years (range: 12-21 years) were evaluated. The mean follow-up period was 35.8 months (range: 12-60 months). The mean length of stay in hospital was 8.7 days (range: 7-10 days). The mean operating time was measured as 200 minutes. Hypotensive analgesia was administered during the operation, and the mean blood transfusion was 650 cc (range: 400-1000 cc).

On the standing anteroposterior radiographs taken preoperatively, the Cobb angle of the major curvatures was calculated as mean 50.83˚ (range: 30˚-72˚). Postoperatively, the mean 68.8% correction at 15.77˚ was determined. At the final follow-up examination, a correction loss of mean 3.3% (range: 0% to 6.8%) was observed.

The age and gender of the patients, the curvature classification according to the Lenke and King classification systems, the preoperative degree of curvature, the postoperative degree of curvature, the correction rates, the degree of curvature at the final follow-up examination, the correction loss rates, follow-up periods, kyphosis angle and the SRS scores are presented in Table 1.

**Table 1:** Summary of all results M: Male, F: Female, SRS: Scoliosis Research Society

	Age	Sex	Fusion Level	Lenke Classification	King Classification	Cobb Angle			Intraoperative Correction	Loss of Correction	Follow-up (years)	Kyphosis Angle (T5-T12)		SRS Score
						Preoperative	Postoperative	Last Follow-up				Preoperative	Postoperative	
Case 1	14	F	T5-L2	1A(-)	Type 3	56	15	16	73.2%	1.7%	3	10	22	3.3
Case 2	12	F	T7-T11	1BN	Type 2	46	16	16	65.2%	------	3	20	28	4.0
Case 3	15	M	T6-T12	1BN	Type 2	48	16	18	66.6%	3.5%	4	20	30	4.1
Case 4	17	F	T11-L4	5CN	Type 1	50	18	20	64%	4%	5	38	24	3.5
Case 5	19	F	T7-T12	1BN	Type 2	40	16	16	60%	------	3	40	25	3.8
Case 6	14	F	T8-L2	1BN	Type 2	52	15	17	71.1%	3.8%	4	35	20	4.1
Case 7	16	F	T6-T11	1BN	Type 2	54	19	21	64.8%	3.7%	4	19	24	3.6
Case 8	15	M	L1-L5	5BN	Type 1	30	18	18	40 %	------	4	26	24	3.5
Case 9	21	F	T6-L1	1AN	Type 2	60	23	23	61.6%	------	3	20	28	3.8
Case 10	19	F	T5-T12	1BN	Type 2	60	20	20	66.6%	------	4	19	24	4.0
Case 11	16	F	T5-T11	1BN	Type 2	48	15	15	68.7%	------	3	22	27	3.6
Case 12	13	M	T5-T12	1BN	Type 2	44	12	15	72.7%	6.8%	2	33	20	4.1
Case 13	16	F	T6-L2	1AN	Type 3	72	19	20	87.5%	1.3%	2	19	26	3.7
Case 14	14	F	T5-T12	1BN	Type 3	53	16	17	69.8%	1.8%	3	10	24	3.7
Case 15	18	F	T10-L3	5C(+)	Type 3	46	7	7	84.7%	------	1	42	23	4.2
Case 16	17	F	T4-T12	1BN	Type 2	46	16	16	65.2%	------	2	12	20	4.5
Case 17	17	M	T9-L3	5CN	Type 3	52	15	15	71.1%	------	1	32	20	3.8
Case 18	16	F	T7-L1	1AN	Type 2	58	8	8	86.2%	------	3	36	26	3.7
Mean	16.05					50.83	15.77		68.8%	3.3%	3	25.16	23.61	3.83

Throughout the follow-up period, no acute or late-term infection was seen in any patient. No neurological deficits were determined in any patient. Pseudoarthrosis was not seen at the end of the follow-up period. There was no need for revision surgery in any patient.

## Discussion

The results of this study showed that 68.8% correction was obtained during surgery with the intraoperative temporary traction method from the posterior, and no complications were seen in any patient. This technique can be used together with all screw and rod systems. After the placement of pedicle screws on both the convex and concave sides of the curvature, this new application is based on the traction of the wire placed on the screws on the convex side of the curve. As the compressive force is created on the convex side, it is neurologically safe, and as it is applied gradually, compression can be applied safely as far as desired accompanied by neuromonitorization. The instruments used are those currently used in deformity surgery and are therefore readily available. 

A limitation of this technique is that despite correction of approximately 60% in the coronal plane, there is no evident correction in the sagittal plane. However, this can be overcome by applying the de-rotation maneuver with the rod placed on the concave side. As the intraoperative temporary traction method from the posterior allows the desired amount of compression to be applied, it is a new method that is helpful in compression and adjusting the distraction in pedicle screw systems.

There are various reduction techniques in scoliosis surgery. In 1999, Delorme described the rod translation maneuver technique, in which the instrumented vertebrae are gradually translated toward the rod using specialized instruments such as the persuader or with pedicle screw extensions [4].

The cantilever bending technique was described by Chang in 2003. This was developed to correct the deformity in the coronal plane using a strong corrective force. A pre-bent rod is locked to the convex side pedicle screws, and then two long, lever arms in the coronal plane, as in situ benders, are secured to the convex side of the rod in the coronal plane. As the lever arms are drawn together, a strong corrective force is generated to correct the curve in the coronal plane [5].

In 2004, Lee described direct vertebral rotation (DVR) to effectively correct a substantial amount of rotational malalignment in scoliosis. DVR was introduced as a procedure applied together with the rod rotation maneuver. Vertebral rotation is corrected by the application of a posterior force in the direction contralateral to the deformity. After the application of pedicle screws and rod rotation to correct the coronal and sagittal deformities, torque is applied to the pedicle screws using long screw derotators on both the concave and convex sides of the curvature. The spinous process is then rotated to the convexity of the curve at the apical and juxta-apical vertebrae [6].

The vertebral co-planar alignment technique was described by Vallespir in 2008. In this technique, the coronal and sagittal profiles are simultaneously recreated together with rotational deformity correction using slotted longitudinal tubes attached to pedicle screws, which are aligned linearly [7].

Ucar described a convex rod rotation technique in 2014, in which the pedicle screws are inserted on the convex side. Following rod placement on the convex side, the rod is rotated toward the convexity of the curve. Screw insertion on the concave side is said to be easier following the rotation maneuver [8].

Compared to other systems, the system described in the current study has some advantages. Although not based on statistical data, the operating time was subjectively seen to be shorter. The most important advantage of the technique is that as the correction can be made gradually and as far as desired, the potential neurological deficits after correction can be controlled. In addition, as the movement of the traction wire is known, distraction at the length desired and compression amounts can be calculated.

## Conclusions

The results of this study showed that no complications were seen in any patient. The most important feature of the technique is that it is neurologically safe, and as it is applied gradually, compression can be applied safely as far as desired accompanied by neuromonitorization. This technique can be considered a simple and safe alternative method for correction in scoliosis surgery.
